# Relationship Between Quorum Sensing and Secretion Systems

**DOI:** 10.3389/fmicb.2019.01100

**Published:** 2019-06-07

**Authors:** Rocio Trastoy Pena, Lucia Blasco, Antón Ambroa, Bertha González-Pedrajo, Laura Fernández-García, Maria López, Ines Bleriot, German Bou, Rodolfo García-Contreras, Thomas Keith Wood, Maria Tomás

**Affiliations:** ^1^Deapartamento de Microbiología y Parasitología, Complejo Hospitalario Universitario A Coruña (CHUAC), Instituto de Investigación Biomédica (INIBIC), Universidad de A Coruña (UDC), A Coruña, Spain; ^2^Departamento de Genética Molecular, Instituto de Fisiología Celular, Universidad Nacional Autónoma de México, Mexico City, Mexico; ^3^Departamento de Microbiología y Parasitología, Facultad de Medicina, Universidad Nacional Autónoma de México, Mexico City, Mexico; ^4^Department of Chemical Engineering, Pennsylvania State University, University Park, PA, United States

**Keywords:** quorum, secretion, virulence, motility, competence

## Abstract

Quorum sensing (QS) is a communication mechanism between bacteria that allows specific processes to be controlled, such as biofilm formation, virulence factor expression, production of secondary metabolites and stress adaptation mechanisms such as bacterial competition systems including secretion systems (SS). These SS have an important role in bacterial communication. SS are ubiquitous; they are present in both Gram-negative and Gram-positive bacteria and in *Mycobacterium* sp. To date, 8 types of SS have been described (T1SS, T2SS, T3SS, T4SS, T5SS, T6SS, T7SS, and T9SS). They have global functions such as the transport of proteases, lipases, adhesins, heme-binding proteins, and amidases, and specific functions such as the synthesis of proteins in host cells, adaptation to the environment, the secretion of effectors to establish an infectious niche, transfer, absorption and release of DNA, translocation of effector proteins or DNA and autotransporter secretion. All of these functions can contribute to virulence and pathogenesis. In this review, we describe the known types of SS and discuss the ones that have been shown to be regulated by QS. Due to the large amount of information about this topic in some pathogens, we focus mainly on *Pseudomonas aeruginosa* and *Vibrio* spp.

## Introduction

Microorganisms coexist in competitive environments with other species, and they must develop different survival strategies to compete for space, nutrients and ecological niches. Bacteria have developed several molecular mechanisms that enable them to survive under stress conditions in different environments. The general stress response (RpoS) (Battesti et al., 2011), tolerance to reactive oxygen species (ROS) (Zhao and Drlica, 2014; Van den Bergh et al., 2017), energy metabolism (cytochrome *bd* complex) (Korshunov and Imlay, 2010) and Tau metabolism (Javaux et al., 2007), drug efflux pumps (Blanco et al., 2016), SOS response (Baharoglu and Mazel, 2014), (p)ppGpp signaling under starvation conditions (Hauryliuk et al., 2015), toxin-antitoxin (TA) systems (Wood et al., 2013) and quorum sensing (QS), which we will discuss in detail in this review, are the main molecular mechanisms of tolerance and bacterial persistence (Harms et al., 2016; Trastoy et al., 2018).

Quorum sensing acts by monitoring cell density through chemical signals that allow communication between bacteria in order to regulate the expression of genes involved in virulence, competition, pathogenicity and resistance (Nealson et al., 1970; Hawver et al., 2016; Paul et al., 2018). In general, QS systems are species-dependent and contribute to processes such as cell maintenance, biofilm formation and horizontal gene transfer. QS also plays a role in other events involving the synchronization of the whole population such as antibiotic production (Abisado et al., 2018), natural competence (Shanker and Federle, 2017), sporulation (Rai et al., 2015) and the expression of secretion systems (SS). In this review, we will focus on the relationship between QS networks and SS in two important bacterial pathogens *Pseudomonas aeruginosa* and *Vibrio* spp.

## QS Network

To explain the structure and functioning of the QS network, we will focus on Gram-negative bacteria, in which the signaling pathways are better described. In general terms, QS systems are composed of synthase proteins that produce QS signals, QS signals, and response regulators that bind QS signals and reprogram gene expression (Ng and Bassler, 2009). *N*-acyl homoserine lactones (AHLs) are the most common QS signals in Gram-negative bacteria (Geske et al., 2008). Other QS signals include autoinducer-2 (AI-2) in *Vibrio harveyi* (Surette et al., 1999), PQS (*Pseudomonas* quinolone signal) (Pesci et al., 1999), DSF (diffusible signaling factor) in *Xanthomonas campestris* (Barber et al., 1997), indole in *Escherichia coli* (Lee and Lee, 2010), and PAME (hydroxyl-palmitic acid methyl ester) in *Ralstonia solanacearum* (Flavier et al., 1997). The LuxI/LuxR QS system of *Vibrio fischeri* is the prototypical model system for Gram-negative bacteria (Engebrecht et al., 1983; Engebrecht and Silverman, 1984). Homologs of *luxI* (which encode synthase proteins) and *luxR* (which encode response regulators) are present in many bacteria (Case et al., 2008). AHL signals are produced inside the cell and most of them are transported freely to the local environment. When the concentration of AHL reaches a certain level outside of the cell, the molecule re-enters the cell (or binds surface receptors) and binds/activates the LuxR-type receptor to alter gene expression. AHL signals with small structural differences are involved in the process of gene regulation (Fuqua et al., 1994; Whiteley et al., 2017; Paul et al., 2018).

*Pseudomonas aeruginosa* possesses three well-known QS systems: LasI/LasR, RhlI/RhlR, and PQS (*Pseudomonas* quinolone signal)/PqsR (MvfR). The Las system consists of LasI, a synthase protein which produces the AHL *N*-(3-oxododecanoyl)-L-homoserine lactone (3O-C12-HSL), and LasR, the transcriptional regulator (Seed et al., 1995; Stintzi et al., 1998; Kariminik et al., 2017). Likewise, the RhlI/RhlR system produces the N-hexanoyl-L-homoserine lactone (C4-HSL) signal and the RhlR transcriptional regulator. Finally, the PQS system comprises 2-heptyl-3-hydroxy-4(1H)-quinolone (PQS signal) and the PqsR (MvfR) receptor (Xiao et al., 2006; Jimenez et al., 2012). In 2016, James and collaborators, analyzed the role of a new binding receptor for PQS signals, i.e., MexG, an inner membrane protein of the *mexGHI-opmD* operon and a component of a resistance-nodulation-cell division (RND) efflux pump (Hodgkinson et al., 2016).

Quorum quenching (QQ) enzymes have also been shown to be important in the functioning of QS systems (Zhang and Dong, 2004; Dong et al., 2007; Bzdrenga et al., 2017). Our research group has recently described a new QQ enzyme (AidA) which participates in the QS network in *Acinetobacter baumannii* clinical strains (Lopez et al., 2017b, 2018).

## Secretion Systems

**FIGURE 1:**
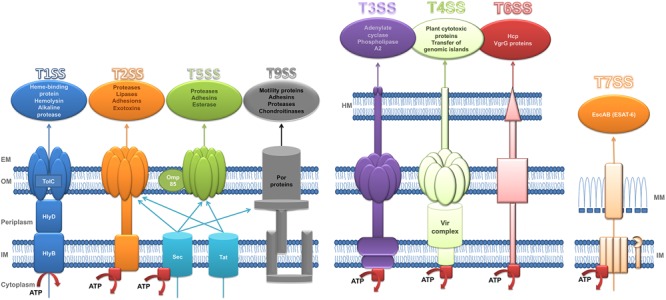
Structure of secretion systems. Schematic representation of secretory systems: type I (T1SS), type II (T2SS), type V (T5SS), type IX (T9SS), type III (T3SS), type IV (T4SS), type VI (T6SS), and type VII (T7SS). The type I pathway is exemplified by hemolysin A (HlyA) secretion in *E. coli* where TolC, HlyD, and HlyB are the three components which constitute the channel to transport HlyA to extracellular space. Sec (general secretion route) and Tat (twin-arginine translocation pathway) transfer the substrates of T2SS and T5SS across the inner membrane. Sec also participates in the transport of T9SS substrates across de inner membrane. T9SS is also called Por Secretion System (PoSS). T4SS is represented by VirB/D system of *Agrobacterium tumefaciens*. The T7SS is based on a system in Mycobacteria. Red squares represent the ATPases. HM, host membrane; EM, extracellular medium; OM, outer membrane; IM, inner membrane; MM, mycomembrane. Substrates secreted by each secretory system are included in a circle in the top of the figure. Adapted from Tseng et al. (2009).

Bacterial pathogens secrete proteins through their cell membranes in a fundamental process that enables them to attack other microorganisms, evade the host immune system, produce tissue damage and invade the host cells. Secreted proteins can act as virulence factors that generate toxic products to the host cells and may also facilitate adhesion to these cells. Translocation of proteins across the phospholipid membranes is carried out by several types of SS (Green and Mecsas, 2016). SS play a significant role in bacterial communication. To date, 8 types of SS (T1SS, T2SS, T3SS, T4SS, T5SS, T6SS, T7SS, and T9SS) have been made defined on their structure, composition and activity (Figure 1). These differences can be attributed to the differences between Gram-negative and Gram-positive bacteria (Desvaux et al., 2009; Sato et al., 2010; Costa et al., 2015). The characteristics of each type of SS are described in detail below.

### T1SS

The type I secretion system is widely distributed in Gram-negative bacteria such as *P. aeruginosa, Salmonella enterica, Neisseria meningitidis*, and *E. coli* (Thomas et al., 2014).

The type I secretion system (T1SS), which has three structural elements (ABC transporter protein, a membrane fusion protein and an outer membrane factor), can transfer substrates across both bacterial membranes in Gram negative bacteria in a one-step process (Green and Mecsas, 2016). T1SS uses proteins as substrates, e.g., proteases and lipases of different sizes and with different functions; these proteins have a C-terminal uncleaved secretion signal which is recognized by the ABC transporter protein to form the translocation complex (Delepelaire, 2004; Kanonenberg et al., 2013).

There are two systems described so far that regulate the expression and secretion of substrates of T1SS, the Has system of *S. marcescens* and *P. aeruginosa*, and the hemolysins of *Vibrio cholerae, N. meningitidis* and in particular of uropathogenic *E. coli* (Thomas et al., 2014).

### T2SS

The type II secretion system (T2SS), which is conserved in most Gram negative bacteria, is responsible for secreting folded proteins from the periplasm. These proteins are first transported through the IM by the general secretory (Sec) or twin-arginine translocation (Tat) pathways, and then secreted from the periplasm into the extracellular medium by the T2SS (Nivaskumar and Francetic, 2014; Green and Mecsas, 2016).

The Sec pathway consists of three structural parts: a protein targeting component, a motor protein and a membrane integrated conducting channel called SecYEG translocase. This mechanism transports unfolded proteins with a hydrophobic sequence at the N-terminus. Moreover, the secreted protein either remains in the periplasm or is transported to the extracellular space. The proteins may contain a SecB-specific signal sequence for transport to the periplasm or the extracellular milieu; however, if it has the signal recognition particle (SRP) signal it can follow the SRP pathway and remain in the inner membrane (Green and Mecsas, 2016; Tsirigotaki et al., 2017).

By contrast, the Tat secretion pathway consists of 2–3 subunits, TatA and TatB, which form a unique multifunctional protein in Gram-positive bacteria, and TatC. This mechanism translocates folded proteins with a twin-arginine motif. In Gram-positive bacteria, most proteins are transported out of the cell, while in Gram-negative bacteria the protein can remain in the periplasm or it can be translocated to the extracellular space by the T2SS (Patel et al., 2014; Green and Mecsas, 2016).

The T2SS, a complex structure composed of 15 proteins, named general secretion pathway proteins (Gsp) in *E. coli* (Korotkov et al., 2012), Eps in *V. cholera* (Abendroth et al., 2009; Sloup et al., 2017) and Xcp in *P. aeruginosa* (Filloux et al., 1998; Robert et al., 2005), has a wide range of substrates with diverse functions, although all share one feature, an N-terminal signal which enables them pass to the periplasm via the Sec or Tat secretion mechanisms (Nivaskumar and Francetic, 2014; Green and Mecsas, 2016).

The main function of the T2SS is to acquire nutrients (Nivaskumar and Francetic, 2014). It is responsible for secreting numerous exoproteins, most of which are hydrolytic enzymes and other proteins such as toxins, adhesins and cytochromes that have various roles in respiration, biofilm formation and motility (Nivaskumar and Francetic, 2014). The T2SS has been described in various environmental strains and also human pathogens such as *V. cholera* (Overbye et al., 1993), *P. aeruginosa, Aeromonas* sp. and enterotoxigenic *Escherichia coli* (ETEC) (Nivaskumar and Francetic, 2014).

### T3SS

The type III secretion system (T3SS) or injectisome, is a double-membrane-embedded apparatus found in multiple pathogenic Gram-negative bacteria such as *Salmonella* spp., *Yersinia* spp., enteropathogenic and enterohemorrhagic *E. coli, Shigella* spp. and *Pseudomonas* spp. (Cornelis, 2006; Gaytan et al., 2016; Deng et al., 2017). This complex nanomachine promotes the transfer of virulence proteins called effectors from the bacterial cytoplasm into the eukaryotic cell in a single step (Galan and Waksman, 2018).

The T3SS is composed of approximately 25 proteins assembled in three main structures: the basal body, a set of rings spanning the two membranes of the bacterium; a hollow needle-shaped component through which the semi-unfolded effectors are transported (these first two structures are collectively called “needle complex”); and the translocon, made up of a hydrophilic protein that serves as a scaffold for forming a translocation pore, constituted by two hydrophobic proteins, which is inserted into the host cell membrane and through which effectors are directly translocated. A unique set of effectors is delivered by each pathogen, which subverts specific host-cell signaling pathways to allow bacterial colonization (Izore et al., 2011; Notti and Stebbins, 2016; Deng et al., 2017).

The export apparatus associated with the basal body is formed by five poly topic inner membrane proteins that are essential for substrate secretion. This protein complex, together with a cytoplasmic sorting platform and the ATPase complex are responsible for substrate recruitment and classification, and for energizing the secretion process enabling chaperone-effector dissociation and protein unfolding for initial entry into the T3SS central channel that serves as the secretion pathway. These components are highly conserved between different T3SS systems and with the flagella, which is evolutionarily related to the injectisome (Abby and Rocha, 2012; Galan and Waksman, 2018; Lara-Tejero and Galan, 2019).

Several effectors of T3SS have been described such as ExoS, ExoT, ExoU, and ExoY in *P. aeruginosa*; Tir and EspE in *E. coli* and YopE, YopH, YopM, YopJ/P, YopO/YpkA, and YopT in *Yersinia* sp. (Cornelis and Van Gijsegem, 2000).

### T4SS

The type IV secretion system family is found in Gram-negative and Gram-positive bacteria as well as in *Archaea*. T4SS is the most cosmopolitan secretion system and differs from other SS as it is able to transfer DNA in addition to proteins (Cascales and Christie, 2003). More specifically, T4SS is capable of performing contact-dependent secretion of effector molecules into eukaryotic cells, conjugative transfer of mobile DNA elements and also exchange of DNA without any contact with the outside of the cell (Green and Mecsas, 2016; Grohmann et al., 2018). T4SS can be divided on the basis of its functionality into two subfamilies: conjugation systems and effector translocators. Conjugation systems are responsible for the transfer of antibiotic resistance genes and virulence determinants among bacteria. The effector translocators introduce virulence factors into the host cell (Christie, 2016). However, in Gram-negative bacteria T4SS has been divided into two different subfamilies: IVA and IVB. The *E. coli* conjugation apparatuses and VirB/D system of *Agrobacterium tumefaciens* are the models used to study the structure of type IVA of T4SS (Grohmann et al., 2018). The VirB/D apparatus consists of 12 proteins which form a complex envelope-spanning structure that facilitate the translocation function. Two of these proteins, VirB2 and VirB5, make up the pilus, while another three proteins act as ATPases, and VirB1 is a lytic transglycosylase (Costa et al., 2015; Green and Mecsas, 2016). The *Legionella pneumophila* Dot/Icm (Defective for organelle trafficking/Intracellular multiplication) system is the model used to study the IVB subfamily of T4SS (Nagai and Kubori, 2011; Grohmann et al., 2018).

### T5SS

The type V secretion system is unique because its substrates transport themselves across the outer membrane. The substrates use the Sec translocase to pass through the inner membrane to the periplasm space. Various different types of T5SS have been identified: autotransporters (T5aSS), two-partner passenger-translocators (T5bSS), trimeric autotransporters (T5cSS), hybrid autotransporters (T5dSS) and inverted autotransporters (T5eSS) (Henderson et al., 2004; Leo et al., 2012; Rojas-Lopez et al., 2017). In general, the T5SS transports proteins across the asymmetric outer membrane (OM) that contains lipopolysaccharides, through their own C-terminal translocation domain that inserts into the OM as a β-barrel to complete the secretion of the N-terminal passenger domain via the barrel pore. Several periplasmic chaperones also participate in transport through the OM, specifically the β-barrel assembly machinery (BAM complex) and the translocation and assembly module (TAM complex) facilitate protein secretion (Rojas-Lopez et al., 2017).

A T5SS has been described in human pathogens such as *Bordetella pertussis* and *Haemophilus influenzae*, which have two-partner SS and uropathogenic *E. coli*, which has chaperone-usher systems (Costa et al., 2015; Green and Mecsas, 2016).

YadA of *Yersinia enterocolitica* and SadA of *Salmonella* are T5SS type c (Leo et al., 2012). Intimin of *E. coli* and invasin of enteropathogenic *Yersinia* spp. are type Ve SS (Leo et al., 2012).

A self-transporter (T5aSS) (Wilhelm et al., 2007) and three T5bSS: LepA /LepB system (Kida et al., 2008), the CupB system (Ruer et al., 2008) and PdtA/PdtB system (Faure et al., 2014), have been reported in *P. aeruginosa*. In *B. cenocepacia*, four T5SS (Holden et al., 2009) have been found, two with pertactin domains and two with haemagglutinin autotransporters; this last type is also present in *S. maltophilia* (Ryan et al., 2009).

### T6SS

The type VI secretion system is widely represented in Gram-negative bacteria (Coulthurst, 2013; Gallique et al., 2017b). T6SS is an integrated secretion device within the membrane and it transfers substrates, which are toxic effectors to eukaryotic (Pukatzki et al., 2007) and prokaryotic cells (Russell et al., 2014). It plays a crucial role in the pathogenesis and competition among bacteria (Ho et al., 2014; Zoued et al., 2014; Costa et al., 2015; Gallique et al., 2017a). The origin of T6SS is related to bacteriophages (Leiman et al., 2009). T6SS is a huge apparatus and consists of 13 core components organized into a *trans-*membrane complex, a baseplate-like structure at the cytoplasmic face of the inner membrane, and a sheathed inner tube, which is the effector delivery module that is ejected to the target cell. The tube-sheath complex is assembled from the baseplate in the cytoplasm and the hollow tube is built from hexamers of the hemolysin co-regulated protein (Hcp). The sheath contracts and pushes the tube with the associated effectors into targeted cells, using a puncturing mechanism similar to the one used by the contractile tails of phages (Russell et al., 2011, 2014; Cianfanelli et al., 2016; Green and Mecsas, 2016; Galan and Waksman, 2018).

### T7SS

Type VII secretory system has been described in some Gram-positive bacteria such as *Staphylococcus aureus* and in species of *Mycobacterium* and *Corynebacterium.* This SS was reported for the first time in 2003 in *Mycobacterium tuberculosis* and it was called ESX-1 (Stanley et al., 2003), which is an important virulence factor in *M. tuberculosis*. To date, five T7SS have been identified in *Mycobacterium* sp. but the transport mechanisms across the mycobacterial membrane are almost unknown (Costa et al., 2015; Ates et al., 2016; Green and Mecsas, 2016).

Most of the substrates of T7SS belong to EscAB clan which includes six protein families: Esx, PE, PPE, LXG, DUF2563, and DUF2580. ESAT-6 is a *M. tuberculosis* protein which belongs to Esx family and which is secreted with EsxB (CFP-10) (Ates et al., 2016).

### T9SS

The type IX secretion system (T9SS) or Por secretion system (PorSS) is the most recently discovered system (Lasica et al., 2017). Its function is to transport molecules across the outer membrane. Its substrates must include a Sec signal, which allows transfer of proteins through the inner membrane with the aid of the Sec system. The T9SS system has been described in almost all members of the phylum *Bacteroidetes*, but it has mainly been studied in oral pathogens such as *Porphyromonas gingivalis* and *Tannerella forsythia.* In *P. gigivalis*, the T9SS system consists of 16 proteins with structural and functional activity, and another two proteins involved in the regulation of the transport process (Sato et al., 2010; Lasica et al., 2017).

## Regulation of Secretion Systems by Quorum Sensing Networks (Table 1)

**Table 1:** Pathogens and QS elements related to secretion systems.

Type secretion system	SS element	QS regulation	QS element	Microorganisms	References
T1SS	Lip	+	*Swr*	*Serratia liquefaciens*	Riedel et al., 2001
T2SS	Xcp	+	*lasR/lasI rhLR/rhlI*	*Pseudomonas aeruginosa*	Wagner et al., 2003; Michel et al., 2007
			DSF-type	*Xanthomonas* species	Qian et al., 2013
T3SS	LEE operon	+	*luxS*	*Escherichia coli*	Sperandio et al., 1999
		–		*Vibrio parahaemolyticus Vibrio harveyi*	Henke and Bassler, 2004
	ExsA		*lasI*	*Pseudomonas aeruginosa*	Liang et al., 2014
	Yop-Ysc	+	Hfq	*Yersinia pseudotuberculosis Yersinia pestis*	Schiano et al., 2014
T4SS	VirB/D	+	VjbR (LuxR-type QS)	*Brucella abortus*	Arocena et al., 2010; Li et al., 2017
		+	*luxI*	Roseobacter group	Patzelt et al., 2013, 2016
T6SS				*Vibrio alginolyticus*	Yang et al., 2018
	Hcp		HapR and LuxO	*Vibrio cholerae*	Ishikawa et al., 2009; Zheng et al., 2010; Kitaoka et al., 2011; Leung et al., 2011
				*Burkholderia thailandensis*	Majerczyk et al., 2016
			AHL	*Pseudomonas fluorescens*	Gallique et al., 2017b
	TseF		PQS	*Pseudomonas* spp.	Lin et al., 2017
			LasR and MvfR	*Pseudomonas aeruginosa*	Lesic et al., 2009
	VipA,Hcp-1, VipB		AbaR/AbaI	*Acinetobacter baumannii*	Lopez et al., 2017b

### Pseudomonas aeruginosa

#### T1SS

Transcriptional studies in *P. aeruginosa* suggest that in this bacterium T1SS is positively regulated by QS, since the expression of its effector, the alkaline protease AprA, depends on QS. In addition, the genes of the AprA inhibitor *aprI* and the structural genes *aprDEF* also appear to be positively regulated by QS (Hentzer et al., 2003; Schuster et al., 2003; Wagner et al., 2003).

#### T2SS

**FIGURE 2:**
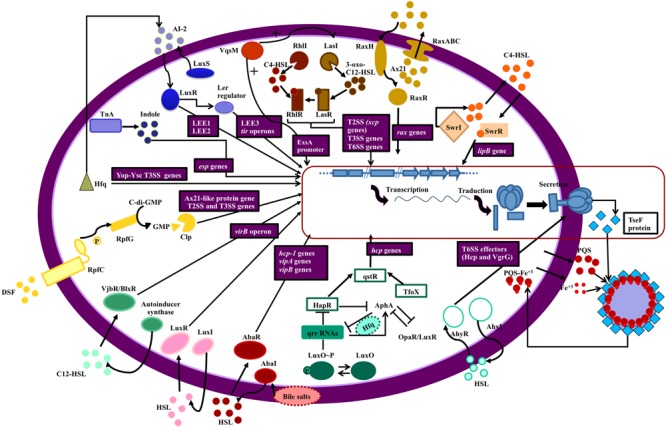
Secretion systems and QS network elements. The figure shows the relationship between QS networks and expression of secretion systems (blue squares). The genes regulated by QS are in purple boxes. Each QS network is represented by a different color. Starting at the top right of the figure: The swr QS system of *S. liquefaciens* controls the *lipB* genes of the T1SS (orange); Ax21 (QS effector) and QS system Rax regulate RaxABC TOSS (T1SS) in gram negative bacteria (ochre); QS (RhlIR and LasIR) regulates expression of T2SS, T3SS, and T6SS in *P. aeruginosa* (brown); VqsM (an AraC family transcription factor) interacts with the LasIR and ExsA promoters (a master regulator of T3SS) in *P. aeruginosa* (dark orange); T2SS is regulated by LuxS/LuxI/AI-2 QS in *E. coli* and indole production by TnA (tryptophanase) regulates *esp* genes expression (T2SS) in this bacterium (blue); in *Yersinia* sp. the Hfq chaperone is connected with QS (AI-2) and regulates the Yop-Ysc type III secretion system (T3SS) (green); in *Xanthomonas* sp. T2SS and T3SS are regulated by DSF (diffusible signal factor) which is a quorum sensing signal (yellow); T4SS (*virB* operon) is regulated by VjbR (LuxR like protein) and LuxI in *Brucella* (turquoise) and *Roseobacter* (pink), respectively; a connection between *Acinetobacter baumanii* QS (AbaI/AbaR, controlled by bile salts) and T6SS has been established (maroon); in *Vibrio* sp. there is a complex network which relates QS (LuxO/HapR/TfoX) with T6SS (aquamarine); AhyRI (a QS network) in *Aeromonas* sp. and *P. atrosepticum* is involved in Hcp and VgrG secretion (sky blue) and finally, iron is transported across the cell membrane accompanied by PQS, a quorum sensing signal in *P. aeruginosa*, and this process depend on Tse a substrate of T6SS, which binds to OMVs (outer membrane vesicles) containing PQS- Fe^3+^.

Three T2SS systems, the Xcp, Hxc and Txc systems, have been described in *P. aeruginosa*. The first of these, Xcp, secretes the QS regulated virulence factors elastase A and B (LasA and LasB) as well as the exotoxin A (ExoA) and it is itself positively regulated by QS (Figure 2). Accordingly, recently it was demonstrated by ChIPseq analysis that MvfR (the receptor of the PQS autoinducer) is able to directly bind *xcpQ-xcpP-xcpR* regions and this is related to their induction in the presence of MvfR (Maura et al., 2016).

The second T2SS, Hxc, is regulated by the availability of phosphate and secretes LapA a low-molecular weight alkaline phosphatase (Wagner et al., 2003; Michel et al., 2007). Two genes, *xphA* and *xqhA*, which encode the PaQa subunit of the Xcp functional hybrid system, have been described. These genes, which are located outside the *xcp* locus, are regulated by environmental conditions but not by QS, in contrast to what occurs with the rest of the Xcp system (Michel et al., 2007, 2011). In contrast to the first two systems, the third system Txc has just recently been described and so far only identified in a region of genome plasticity of the strain PA7; it is regulated by a two component system (TtsSR) and secretes the chitin binding protein CpbE (Cadoret et al., 2014).

#### T3SS

Current evidence suggest that as in *Vibrio* spp., QS in *P. aeruginosa* negatively regulates the expression of T3SS, specifically the RhlI/RhlR system, as transcription of the T3SS genes and secretion of ExoS increase significantly in a *rhlI* mutant and return to basal levels on the addition of exogenous C4-HSL (Bleves et al., 2005; Kong et al., 2009; Figure 2). In agreement, the expression of *exoS* is also negatively regulated by QS, specifically by the RhlI/RhlR system, as well as by the stationary phase sigma factor RpoS (Hogardt et al., 2004).

The fact that the T3SS genes do not appear to be repressed by QS in some global transcriptomic studies with mutants may be explained by the presence of high calcium concentrations in the media, or by the lack of resolution of DNA microarrays (Hentzer et al., 2003; Schuster et al., 2003). More striking is the fact that some QS inhibitors like 6-gingerol and coumarin inhibit rather than increase the expression of T3SS (Zhang et al., 2018). Nevertheless, these studies were done in the presence of high calcium, and QS-independent inhibition of T3SS has not been ruled out. Moreover, a recent study in the PA01 strain, using a *lasR rhlR* double mutant, demonstrated that it remains virulent in a murine abscess model, despite that it does not produce QS-dependent virulence factors and that the secretion of ExoT and ExoS is fully functional in this mutant. Hence the authors hypothesized that T3SS is the cause of the remaining virulence (Soto-Aceves et al., 2019).

The *P. aeruginosa* QS network and its T3SS are also related by the fact that VqsM, an AraC-family transcription factor, binds to both the promoter region of *lasI* and the promoter of *exsA*, which encodes a master regulator of the T3SS, regulating both mechanisms (Liang et al., 2014; Figure 2).

#### T6SS

The T6SS is involved in iron transport, and a connection has been observed between T6SS and QS through the TseF protein, which is a substrate of T6SS and interacts with PQS (Lin et al., 2017; Figure 2).

In *P. aeruginosa*, three loci which encode T6SS have been found to be regulated by QS proteins (LasR and MvfR) (Lesic et al., 2009). Expression of the second loci, H2-T6SS, is regulated by the Las and Rhl QS systems in PAO1 strains (Sana et al., 2012; Figure 2) and by the direct binding of MvfR in PA14 (Maura et al., 2016).

### *Vibrio* sp.

#### T2SS

The formation of biofilms has multifactorial regulation in *V. cholerae* as in other pathogens. The QS network controls directly biofilm production which is related to type II secretion system in *V. cholerae* (Teschler et al., 2015). Several proteins such as RbmA, RbmC and Bap1, which are involved in the formation of biofilms, are transported by T2SS. In addition, mutant strains with inactivated T2SS have reduced biofilm formation (Johnson et al., 2014; Teschler et al., 2015).

#### T3SS

In *V. parahaemolyticus* and *V. harveyi* (unlike in *E. coli*), both the HAI-1 and AI-2 QS systems inhibit the expression of T3SS genes (Henke and Bassler, 2004). QS also represses T3SS during *V. harveyi* infections of gnotobiotic brine shrimp (Ruwandeepika et al., 2015). Waters et al. (2010) have described the regulatory pathway by which QS controls T3SS. At low cell density when LuxR is repressed, which entails the derepression of two promoters of the *exsBA* operon and the *exsA* operon, ExsA activates the expression of genes that encode the structural proteins of the type III secretion system. However, when the cell density is high, LuxR directly represses transcription of the PB promoter, preventing the production of ExsA and consequently decreasing the expression of structural genes of T3SS (Waters et al., 2010; Ball et al., 2017). OpaR inhibits the T3SS1 in *V. parahaemolyticus* which is the most important factor in its cytotoxicity (Gode-Potratz and McCarter, 2011).

#### T6SS

Several researchers have demonstrated the regulation of T6SS by QS networks in *Vibrio* spp. We present the main findings in this field here. In *V. alginolyticus*, activation of T6SS and the QS network has been found to be coordinated by the serine/threonine kinase PpkA cascade (Yang et al., 2018). PpkA2 is autophosphorylation and it transfers the phosphate group to VstR. Phosphorylated VstR promotes the expression of both of the T6SS in *V. alginolyticus* through the inhibition of LuxO activity, which acts to impede the expression of LuxR, a promoter of the T6SS. LuxR inhibits the expression of the first T6SS (T6SS1) or promotes the expression of the second T6SS (T6SS2) (Yang et al., 2018).

At low cell population density, LuxO is phosphorylated, which activates the expression of specific small regulatory RNAs (sRNAs) in conjunction with alternative sigma factor σ^54^ (Sheng et al., 2012). sRNAs inhibit the expression of LuxR with the help of RNA chaperone Hfq (Liu et al., 2011). However, at high cell population density, LuxO is dephosphorylated turning off the transcription of the sRNAs and allowing the translation of LuxR (Waters and Bassler, 2005; Milton, 2006). Sheng et al. (2012) also demonstrated that the expression of the *hcp* T6SS gene is growth phase-dependent and the QS regulators controls the haemolysin co-regulated protein, which is one of the main proteins of the T6SS functioning as an effector of the system and/or an effector binding protein (Figure 2). The phosphatase PppA also acts on the QS (modulating the transcription of LuxR) and the expression and secretion of *hcp1* and *hcp2* (Sheng et al., 2013). It is important to highlight that PppA permits the cross-talk between the two T6SS in *V. alginolyticus* (Sheng et al., 2013).

Rpo N (σ^54^) collaborates with QS in the regulation of T6SS genes. It is involved in the regulation of the expression of *hcp* and *vgrG3* operons that encode T6SS secreted molecules, but does not control the genes that encode the structural and sheath components of T6SS (Ishikawa et al., 2009; Dong and Mekalanos, 2012).

There are a few more studies in *V. cholerae* related to this topic than other species. Two QS autoinducers, CAI-I (cholerae autoinducer) and AI-2 (autoinducer-2), co-operate to control the gene expression depending on the cell density (Ng and Bassler, 2009). Two enzymes are necessary for the biosynthesis of these autoinducers: CqsA and LuxS, respectively (Schauder et al., 2001; Miller et al., 2002; Chen et al., 2002; Higgins et al., 2007). These signal molecules are detected by two sensor kinases, LuxQ (sensor of CAI-I) and CqsS (sensor of AI-2). Both pathways merge on LuxU, a phosphotranfer protein. At low cell density (LCD), the two sensor kinases phosphorylate LuxU due to the absence of their respective autoinducers. There are two histidine kinases which also contribute to the phosphorylation of LuxU: VpsS and CqsR (Jung et al., 2015). Then, LuxU transfers the phosphorylate group to a DNA-binding response regulator protein called LuxO. Phosphorylated LuxO activates the expression of sRNA molecules (known as qrr1-4) when the cell density is low thanks to the interaction with the alternative sigma factor σ^54^ (Freeman et al., 2000; Lenz et al., 2004). In conjunction with the RNA-binding protein Hfq, LuxO represses the expression of HapR (Lenz et al., 2004), a TetR-family global transcriptional regulator which acts on QstR (Tsou et al., 2009; Shao and Bassler, 2014; Watve et al., 2015; Figure 2). HapR is accumulated when the cell density is high (Lenz et al., 2004) because LuxO is not phosphorylated and transcription of the sRNAs is blocked. QstR is a master regulator of the T6SS belonging to the LuxR-type family of regulators (Jaskólska et al., 2018). QstR binds to the promoter region of the T6SS cluster inducing the expression of the genes. The regulation of the T6SS by cAMP-CRP pathway is not clear, but it is possible that it influences T6SS genes through regulation of QS and chitin-induced competency (Liang et al., 2007; Blokesch, 2012). It is known that CRP positively regulates T6SS (Ishikawa et al., 2009). Apart from the activation of QstR via QS, it is also regulated by chitin and arabinose (Lo Scrudato and Blokesch, 2012, 2013).

The expression of the three T6SS gene clusters in *V. cholerae* requires TfoX, CytR, HapR, and QstR for the highest level of expression (Watve et al., 2015). CytR and TfoX are required for the expression of the T6SS genes but their regulatory effects are only mediated by QstR (Figure 2).

### Other Pathogens

#### T1SS

The *swr* QS system, which controls swarming motility, regulates the Lip secretion system, a T1SS responsible for the secretion of lipases, metalloproteases and S-layer proteins in *Serratia liquefaciens* MG1 (Riedel et al., 2001). The *swr* QS system consists of SwrI, which synthesizes C4-HSL, and SwrR, which regulates gene transcription after binding the diffusible signal C4-HSL. QS-mediated regulation of *lipB*, which encodes the LipB exporter, was demonstrated in *swrI* mutants with *luxAB* insertions, in which the level of secreted proteins was lower (Riedel et al., 2001; Figure 2). Other relationships between T1SS and QS have also been observed. The rice pathogen recognition XA21 receptor recognizes a sulphated peptide (axY^S^22) derived from the Ax21 protein (activator of XA21-mediated immunity) and confers resistance to *Xanthomonas oryzae* strains. Ax21 may have a key biological role because it is conserved in *Xanthomonas* spp., *Xylella fastidiosa*, and *Stenotrophomonas maltophilia*. Ax21 requires RaxABC TOSS (type I secretion system) for secretion and activity. The expression of *rax* genes which encode T1SS has been demonstrated to be QS-dependent due to the cell-density dependency (Han et al., 2011). These data indicate that Ax21 could have a role as a signaling molecule and a direct relationship between the QS network and T1SS is established (Figure 2; Lee et al., 2006).

#### T2SS

In *Xanthomonas* species, QS is mediated by the diffusible signal factor (DSF). A proteomic analysis conducted in 2013 revealed 33 proteins that are controlled by DSF. Their putative functions are associated with QS and include cellular processes, intermediary metabolism, oxidative adaption, macromolecule metabolism, cell-structure, protein catabolism, and hypothetical functions (Qian et al., 2013). In this study, it was observed that three genes encoding T2SS-dependent proteins and one gene which encodes Ax21 (activator of XA21-mediated immunity)-like protein are regulated by QS and are essential for virulence-associated functions, including extracellular protease, cell motility, antioxidative ability, extracellular polysaccharide biosynthesis (EPS), colonization, and biofilm (Qian et al., 2013; Figure 2).

#### T3SS

The relationship between QS and T3SS in *E. coli* was first demonstrated by Sperandio et al. (1999), who showed that expression of the locus of enterocyte effacement (LEE) operons that encode the T3SS is activated by QS in both enterohemorrhagic (EHEC) and enteropathogenic (EPEC) *E. coli* due to transcriptional control of the LEE operons by LuxS, which directly activates the LEE1 and LEE2 operons and indirectly activates (via the Ler regulator) the LEE3 and *tir* operons (Figure 2). These researchers proposed that activation of the T3SS by the AI-2 autoinducer synthesized by commensal *E. coli* resident in the large intestine could explain the high infectivity of *E. coli* O157: H7, which has an infectious dose of about 50 bacterial cells (Sperandio et al., 1999).

The major virulence factors of EHEC and EPEC are intimin (T5eSS), Tir (the receptor for intimin) and the three secreted proteins EspA, EspB and EspD. T3SS functions in the secretion of the Tir and Esp proteins. The LuxR-type response regulator SdiA negatively regulates the expression of EspD and intimin in the same bacterium, indicating multifactorial regulation of the T3SS by bacterial QS signals (Kanamaru et al., 2000).

Indole, which is produced by tryptophanase (TnA) in enteric bacteria and reaches high concentrations in the gut, is another signaling molecule that influences expression of T3SS in *E. coli* (Lee et al., 2007, 2008). Indole increases the production and secretion of the translocators EspA and EspB in EHEC O157:H7 (Hirakawa et al., 2009; Figure 2); hence, indole promotes the development of attaching and effacing (A/E) lesions in HeLa cells.

The involvement of the RNA chaperone protein Hfq, which also participates in QS, in T3SS expression was demonstrated in *Yersinia pseudotuberculosis* and *Yersinia pestis* (Schiano et al., 2014; Figure 2). Moreover, Schiano et al., 2014 have demonstrated the regulation of T3SS by QS through virulence regulators LcrF and YmoA in *Y. pseudotuberculosis* (Amy, 2018).

In *Aeromonas hydrophila*, an unique QS system, encoded in *ahyR/ahyI* loci, has been described (Vilches et al., 2009; Garde et al., 2010). Vilches et al. (2009) have used the *A. hydrophila* AH-3 strain to study the T3SS regulation. AH-3: ahyI and AH-3: ahyR mutants have reduced activity of the *aopN-aopB* promoter (promoter of T3SS components) compared to the wild-type strain (Figure 2). So they concluded that QS could be involved in the positive regulation of the production of the T3SS component in the AH-3 strain (Vilches et al., 2009).

#### T4SS

In *Brucella abortus*, there is a clear relationship between the QS network and T4SS. For the *virB* operon, which encodes the T4SS regulated by VjbR, a LuxR-type QS is responsible for the virulence characteristics of *B. abortus* (Li et al., 2017). The *virB* operon is responsible for establishing the replicative niche of the bacterium once it enters the host cell. The T4SS in *B. abortus*, as in other bacteria, translocates effector proteins into the host cell to avoid the immune defense mechanisms, making it one of the two main virulence factors for *Brucella.* Arocena et al. (2010) described the binding site of VjbR to the *virB* operon (Li et al., 2017). Otherwise, the conjugation process between two members of the *Roseobacter* group mediated by T4SS, encoded in RepABC-type plasmids, is controlled by the QS network. This was demonstrated by construction of *luxI* mutant and the addition of external long chain AHLs, which restored the phenotype (Patzelt et al., 2013, 2016; Figure 2).

#### T6SS

Quorum sensing has been reported to control expression of T6SS toxin-immunity systems in *Burkholderia thailandensis*. Moreover, a new role for T6SS in constraining the proliferation of QS mutants has been described in *B. thailandensis* (Majerczyk et al., 2016). Interestingly, it has been observed that T6SS effectors function as cell-to-cell signals in a *Pseudomonas fluorescens* MFE01 strain lacking the AHL QS pathway (Gallique et al., 2017b).

In *A. hydrophila*, Hcp and VgrG- two of the “core” proteins and also effectors of the T6SS- secretion have been suggested to be regulated by the AhyRI QS regulon (Khajanchi et al., 2009; Figure 2). Finally, our research group has described the association between T6SS machinery and the activation of the QS system by bile salts in *A. baumannii* clinical strains (Lopez et al., 2017a; Figure 2).

#### T7SS

As with other bacteria, *Mycobacterium* spp regulate biofilm formation by QS (Virmani et al., 2018). The second messenger c-di-cGMP, an intracellular signaling molecule, coordinates biofilm production and QS signaling (Sharma et al., 2014). Both *M. tuberculosis* (Kulka et al., 2012) and diverse species of non-tuberculous mycobacteria (*M. smegmatis, M. marinum, M. fortuitum, M. chelonae, M. ulcerans, M. abscessus, M. avium*, and *M. bovis*) produce biofilm depending on certain environmental conditions such as the availability of nutrients or the pH of the medium (Hall-Stoodley et al., 1998; Bardouniotis et al., 2003; Ojha et al., 2005; Marsollier et al., 2007; Johansen et al., 2009; Rhoades et al., 2009).

In the recent work of Lai et al. (2018) it was demonstrated that *the espE, espF, espG*, and *espH* genes, located in the T7SS ESX-1 operon, are crucial for sliding motility and biofilm formation in *M. marinum*. Esp proteins, which regulate substrate transport, are involved also in virulence. This paper clearly demonstrates the role of *M. marinum* T7SS in the production of biofilm which, as already mentioned, is related to QS (Lai et al., 2018).

The T7SS of *S. aureus*, a virulence factors export machinery, plays a key role in the promotion of bacterial survival and long-term persistence of subpopulations of staphylococci. The expression of T7SS is regulated by the bacterial interaction with host tissues (Lopez et al., 2017c) mediated by the secondary sigma factor (σB) (Schulthess et al., 2012). Schulthess et al. (2012) reported that the repression of *esxA* by σB is due to the transcription of *sarA* induced by σB, which leads to a strong repression of *esxA*. The activation of the *esxA* transcript, on the other hand, is stimulated by *arlR*, the response regulator of the ArlRS two-component system, SpoVG, a σ-dependent element, and the Agr quorum detection system (Schulthess et al., 2012). Agr QS system is composed by AIP (self-activating peptide), the inducer ligand of AgrC which is the receptor of the agr signal. In the case of the QS Agr system, the effector of global gene regulation is an important regulatory RNA, RNAIII (Novick and Geisinger, 2008).

#### T9SS

Moreover, an important relationship between T9SS and biofilm formation has been observed in periodontopathogenic pathogens such as *Capnocytophaga ochracea, Porphyromonas* spp., *Fusobacterium* spp. and *Prevotella* spp. (Kita et al., 2016). In the study by Kita et al. (2016), the participation of T9SS in the formation of biofilm of *C. ochracea* is demonstrated. The formation of biofilm of *C. ochracea* is crucial for the development of dental plaque and the same happens with other periodontal pathogens, in which it has also been seen that genes related to T9SS are present. Therefore, the components of the T9SS could be potential targets to inhibit the formation of biofilm and thus avoid the formation of dental plaque (McBride and Zhu, 2013; Kita et al., 2016). However, in depth analysis of the relationship between T9SS and QS network in different pathogens is required.

## Discussion

To date, the T1SS, T2SS, T3SS, T4SS, T6SS, T7SS, and T9SS SS have been found to have important relationships with QS networks. The involvement of the T1SS system (Lip B which is part of the Lip exporter) in the QS network (*swr* quorum system) of *S. liquefaciens* MG1 has been investigated (Riedel et al., 2001). In *P. aeruginosa*, two QS systems (*lasR/lasI* and *rhLR/rhlI*) are linked to T2SS system by microarrays and proteomic studies (Chapon-Herve et al., 1997; Wagner et al., 2003; Michel et al., 2007), and DSF-type systems are also linked to T2SS in *Xanthomonas* species through proteome analysis (Qian et al., 2013). The QS signal AI-2 has been associated with a T3SS system in *E. coli* (Sperandio et al., 1999) and *Vibrio* spp. (Henke and Bassler, 2004). Moreover, this T3SS system has been related to QS proteins in another two pathogens, *P. aeruginosa* and *Yersinia* spp. (Liang et al., 2014; Schiano et al., 2014).

Several T4SS (*virB* operon) are controlled by VjbR protein which is a LuxR-type quorum-sensing regulator in *B. abortus* (Arocena et al., 2010; Li et al., 2017). Moreover, in the *Roseobacter* group, the conjugation of plasmids, which encode T4SS, is QS-controlled and the QS system may detect a broad range of long-chain AHLs at the cell surface (Patzelt et al., 2013, 2016).

There is a wealth of information relating the T6SS to QS in pathogens such as *Vibrio* spp. For example, Hcp and VasH from the T6SS system in *V. cholerae* are involved in QS (Ishikawa et al., 2009; Zheng et al., 2010; Kitaoka et al., 2011; Leung et al., 2011; Yang et al., 2018). For *Pseudomonas* spp,. there are numerous works where the different T6SS are regulated by QS networks (Lesic et al., 2009; Gallique et al., 2017b; Lin et al., 2017). In other pathogens as *Burkholderia thailandensis* (Majerczyk et al., 2016), and in *A. baumannii*, the relationship between QS and SS has begun to be studied (Lopez et al., 2017a).

In *M. marinum*, the relationship between biofilm formation, which is tightly connected with QS, and T7SS, has been demonstrated (Lai et al., 2018). Also in *S. aureus*, the Agr QS network has been related to T7SS (Schulthess et al., 2012). An important relationship between T9SS and biofilm formation has been observed in periodontopathogenic pathogens (Kita et al., 2016). Finally, although the involvement of T5SS secretion system in virulence, motility and competence is well-known, these systems and their association with QS must be studied in greater depth in order to clarify their roles.

Taking *P. aeruginosa* as a reference point, the positive effect of QS in the expression of T1SS and T2SS could be related to the fact that this organism secretes exoproducts that are public goods (proteases and lipases); hence, it is better to produce and secrete these compounds when a high cell density is reached, since these products are costly and the benefits associated to their production are higher at high cell densities. Similarly, the T6SS, which is involved in killing competitors by contact, will be more efficient at high cell densities since the probability of finding target bacteria is higher. In contrast, the T3SS appears to be negatively regulated by QS, and this may be related to its role in an acute infection and its “inhibition by QS” may be a way to facilitate the transition to a chronic infection state. In addition to its well established role in infections, T3SS has a broader ecological role suggested by its role in killing biofilm associated *Acanthamoeba castellanii* amoeba (Matz et al., 2008). Furthermore, it was recently demonstrated that T3SS is susceptible of cheating by mutants that do not produce it, allowing their establishment in infections (Czechowska et al., 2014); hence, the selective forces that act over T3SS are complex.

Therefore, research into the relationship between QS and SS must be further developed in order to better understand human infections.

## Author Contributions

RTP, LB, AA, BG-P, LF-G, ML, IB, and GB developed the redaction of the manuscript, figures and table. RG-C, TW, and MT designed the review, assigned writing tasks to co-authors, contributed to writing and proofread the final version.

## Conflict of Interest Statement

The authors declare that the research was conducted in the absence of any commercial or financial relationships that could be construed as a potential conflict of interest.
